# Genomic risk scores for juvenile idiopathic arthritis and its subtypes

**DOI:** 10.1136/annrheumdis-2020-217421

**Published:** 2020-09-04

**Authors:** Rodrigo Cánovas, Joanna Cobb, Marta Brozynska, John Bowes, Yun R Li, Samantha Louise Smith, Hakon Hakonarson, Wendy Thomson, Justine A Ellis, Gad Abraham, Jane E Munro, Michael Inouye

**Affiliations:** 1 Cambridge Baker Systems Genomics Initiative, Baker Heart Research Institute - BHRI, Melbourne, Victoria, Australia; 2 Childhood Arthritis, Murdoch Children's Research Institute, Melbourne, Victoria, Australia; 3 Department of Paediatrics, The University of Melbourne, Melbourne, Victoria, Australia; 4 Centre for Genetics and Genomics Versus Arthritis, Centre for Musculoskeletal Research, University of Manchester, Manchester, United Kingdom; 5 National Institute of Health Research Manchester Biomedical Research Centre, Manchester Academic Health Science Centre, Manchester University NHS Foundation Trust, Manchester, United Kingdom; 6 Center for Applied Genomics, Children’s Hospital of Philadelphia, Philadelphia, Pennsylvania, United States; 7 Helen Diller Family Comprehensive Cancer Center, Department of Radiation Oncology, University of California San Francisco, San Francisco, California, United States; 8 Perelman School of Medicine, University of Pennsylvania, Philadelphia, Pennsylvania, United States; 9 Murdoch Children's Research Institute, Royal Children's Hospital, Melbourne, Victoria, Australia; 10 Faculty of Health, Centre for Social and Early Emotional Development, Deakin University, Burwood, Victoria, Australia; 11 Cambridge Baker Systems Genomics Initiative, Department of Public Health and Primary Care, University of Cambridge, Cambridge, United Kingdom; 12 Department of Clinical Pathology, University of Melbourne, Melbourne, Victoria, Australia; 13 Paediatric Rheumatology Unit, Royal Children’s Hospital, Melbourne, Victoria, Australia; 14 British Heart Foundation Cardiovascular Epidemiology Unit, Department of Public Health and Primary Care, University of Cambridge, Cambridge, United Kingdom; 15 British Heart Foundation Centre of Research Excellence, University of Cambridge, Cambridge, United Kingdom; 16 National Institute for Health Research Cambridge Biomedical Research Centre, University of Cambridge and Cambridge University Hospitals, Cambridge, United Kingdom; 17 Health Data Research UK Cambridge, Wellcome Genome Campus and University of Cambridge, Cambridge, United Kingdom; 18 The Alan Turing Institute, London, United Kingdom

**Keywords:** arthritis, juvenile, polymorphism, genetic, arthritis, rheumatoid

## Abstract

**Objectives:**

Juvenile idiopathic arthritis (JIA) is an autoimmune disease and a common cause of chronic disability in children. Diagnosis of JIA is based purely on clinical symptoms, which can be variable, leading to diagnosis and treatment delays. Despite JIA having substantial heritability, the construction of genomic risk scores (GRSs) to aid or expedite diagnosis has not been assessed. Here, we generate GRSs for JIA and its subtypes and evaluate their performance.

**Methods:**

We examined three case/control cohorts (UK, US-based and Australia) with genome-wide single nucleotide polymorphism (SNP) genotypes. We trained GRSs for JIA and its subtypes using lasso-penalised linear models in cross-validation on the UK cohort, and externally tested it in the other cohorts.

**Results:**

The JIA GRS alone achieved cross-validated area under the receiver operating characteristic curve (AUC)=0.670 in the UK cohort and externally-validated AUCs of 0.657 and 0.671 in the US-based and Australian cohorts, respectively. In logistic regression of case/control status, the corresponding odds ratios (ORs) per standard deviation (SD) of GRS were 1.831 (1.685 to 1.991) and 2.008 (1.731 to 2.345), and were unattenuated by adjustment for sex or the top 10 genetic principal components. Extending our analysis to JIA subtypes revealed that the enthesitis-related JIA had both the longest time-to-referral and the subtype GRS with the strongest predictive capacity overall across data sets: AUCs 0.82 in UK; 0.84 in Australian; and 0.70 in US-based. The particularly common oligoarthritis JIA also had a GRS that outperformed those for JIA overall, with AUCs of 0.72, 0.74 and 0.77, respectively.

**Conclusions:**

A GRS for JIA has potential to augment clinical JIA diagnosis protocols, prioritising higher-risk individuals for follow-up and treatment. Consistent with JIA heterogeneity, subtype-specific GRSs showed particularly high performance for enthesitis-related and oligoarthritis JIA.

**Key messages**

**What is already known about this subject?**

- The diagnosis of juvenile idiopathic arthritis (JIA) is made purely using history and physical examination.
- No sensitive or specific tests are available to assist clinicians in making the diagnosis.
- JIA has similar genetic architecture to other autoimmune diseases and a strong association with the human leukocyte antigen (HLA) locus.

**What does this study add?**

- Demonstrates genomic machine learning can yield predictive genomic risk scores (GRSs) for JIA.
- Subtype-specific GRSs better capture risk of each subtype separately.
- Subtypes that take the longest to identify or are most common may benefit most from GRSs.

**How might this impact on clinical practice or future developments?**

- These GRSs have the potential to augment current JIA diagnosis protocols, prioritising higher-risk individuals for follow-up and treatment and reducing delays.
- Subtype-specific analyses highlight the potential for genetic studies to better understand heterogeneous diseases such as JIA, potentially paving the way for better disease subtype prediction in general.

## Introduction

Juvenile idiopathic arthritis (JIA) is an autoimmune disease that comprises all forms of arthritis arising before the age of 16 years and persisting for more than 6 weeks.^1^ JIA has a significant impact on quality of life, physical function and future development, and its prevalence is estimated at 0.07 to 4 per 1000 children of European descent.^2–4^ The International League of Associations of Rheumatology (ILAR) classification system recognises seven subtypes of JIA based on the number of joints affected, age of onset and other features.^5^ The aetiology of JIA is not well understood and its diagnosis remains purely dependent on clinical presentations, which can be highly variable between patients. Currently, there are no sensitive or specific tests available to assist clinicians in making the diagnosis.

**Figure 1:**
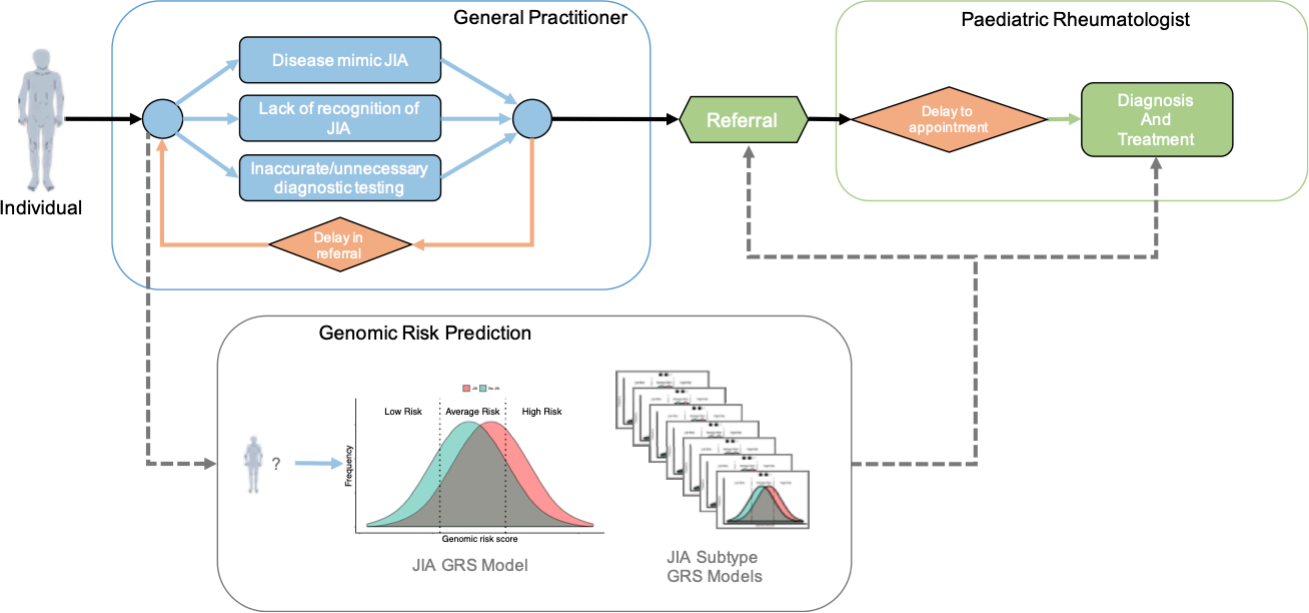
Schematic of a typical clinical path from first symptoms to JIA diagnosis and treatment. Potential informative points are included for JIA genomic risk scores to prioritising higher-risk individuals for referral, follow-up and treatment. GRS, genomic risk score; JIA, juvenile idiopathic arthritis.

Early diagnosis and treatment of JIA is critical as delays increase the risk of prolonged and uncontrolled disease, with consequent poorer long-term outcomes.^6–9^ However, in most cases, general practitioners and paediatricians have limited experience in recognising and diagnosing JIA. This affects time to diagnosis and causes delays in referral and treatment (figure 1). Furthermore, symptomatic children who turn out not to have JIA may be inappropriately referred to paediatric rheumatologists for management,^10–12^ putting undue pressure on clinics and waiting lists. A study in the UK found that the average time from symptom onset to first paediatric rheumatology assessment was ~7 months, with significant variation among JIA subtypes (range of median interval from 6 to 60 weeks), which was due to complex pathways of referral and inappropriate invasive investigations.^13^ Therefore, there is an urgent need for tools that assist clinicians in assessing children who may be JIA cases, and thus reduce the risk of disease complications and poorer long-term health outcomes.

JIA is a complex disease^14^ and susceptibility is due to a complex interaction between genetic and environmental factors. It has been shown that JIA is heritable and that it possesses a genetic architecture similar to other autoimmune diseases, including shared susceptibility genes, mainly in the human leukocyte antigen (HLA) region.^15–17^ For first cousins the recurrence risk has been estimated to be 5.8-fold and for siblings, it has been estimated to be 11.6-fold.^18^ Taken together, it is apparent that genetics have an important aetiological role in JIA, and may have utility in risk prediction, potentially via stratification of JIA cases from non-cases to aid clinical diagnosis.

Genetics is increasingly used to aid risk prediction, diagnosis and earlier treatment of human diseases, with HLA testing for various immune disorders being an example. More recently, the clinical utility of genetic and polygenic risk scores for diverse aetiologies, from coeliac disease to cardiovascular diseases, has come under intense investigation.^19–23^ In coeliac disease, research has shown that a genomic risk score (GRS) based on genome-wide genetic variation (SNPs (single nucleotide polymorphisms)) can accurately predict cases from controls with high specificity and sensitivity, compared with other approaches.^22^ Furthermore, array technologies are relatively affordable with genotyping only needing to be performed once at any point in the lifetime of an individual. GRSs themselves are quantitative measurements of the likelihood that an individual of unknown phenotype has, or will have, a particular disease. Thus, GRSs provide potential advantages in terms of flexibility for clinical translation, as compared with other tests which are temporally variable or offer only a binary ‘susceptible/not susceptible’ output.

This study aims to create a GRS which in-principle could be used to support the current clinical JIA diagnosis practice. We used three large-scale independent cohorts of European ancestry to develop and test a GRS for JIA. Furthermore, we extended the GRS approach to design JIA subtype-specific GRSs, which we externally tested to quantify their potential relative clinical value in supporting each JIA subtype's time to diagnosis.

## Methods

### Phenotype and clinical data

The ILAR classification system^5^ provides generally-accepted guidelines for researchers and clinicians to delineate the seven mutually-exclusive categories of JIA based on the dominant clinical and laboratory features. In this work, the JIA diagnosis for three cohorts (UK, US-based and Australia) was made according to the ILAR revised criteria, and the age of onset of all cases was <16 years old. In the UK cohort,^15^ the JIA cases were obtained from five sources: The British Society for Paediatric and Adolescent Rheumatology National Repository of JIA; a group of UK cases with long-standing JIA described previously;^24^ a cohort collected as part of the Childhood Arthritis Prospective Study;^10^ a cohort of children recruited for the SPARKS-CHARM (Childhood Arthritis Response to Medication);^25^ and an ongoing collection of UK cases from the UK JIA Genetics Consortium. The controls were population-based from the shared UK 1958 Birth cohort and UK Blood Services Common Controls, genotyped as part of the Wellcome Trust Case Control Consortium (WTCCC).^26^

In the US-based cohort, from the Children’s Hospital of Philadelphia (CHOP),^17 27^ the JIA cases were collected from the electronic health records (EHR) completed by the paediatric rheumatology specialist within the Division of Rheumatology and abstracted into a JIA Registry maintained within the Center for Applied Genomics (CAG) at CHOP. Controls were unrelated and disease-free children recruited by the CAG team within the CHOP Healthcare Network. In addition, controls had no history of JIA or other chronic illnesses and were screened as negative for a diagnosis of autoimmune diseases, based on data from CHOP’s EHR and by intake questionnaires obtained by the recruiting staff from the CAG at CHOP.

Finally, in the Australian cohort, from the ChiLdhood Arthritis Risk factor Identification sTudY (CLARITY),^28 29^ all the cases were JIA diagnosed by a paediatric rheumatologist. Incident cases were defined as children recruited within 6 months of diagnosis and prevalent cases were defined as those children diagnosed more than 6 months before recruitment and since 1997. Controls were recruited through the Royal Children’s Hospital Day Surgery Unit. Exclusion criteria for cases and controls were the presence of major congenital abnormalities or illness that would forgo school attendance in the year prior to recruitment.

### Genotype data and quality control

All genotypes included in each cohort were aligned to the GRCh37/hg19 assembly build and passed stringent quality control (QC) measures. Additionally, the QC cohorts were imputed to harmonise and maximise the genetic information across them. All the individuals considered were of European descent and outliers from each cohort were removed to achieve more homogeneous samples.

The initial UK cohort consisted of 2758 cases and 5187 controls. Controls were obtained from the WTCCC, which have been demonstrated to be well-matched to the UK JIA cases,^15 30^ and were genotyped on the Illumina HumanOmniExpress array. For the UK JIA cases, 1670 were genotyped on the Illumina HumanOmniExpress array and 1088 on the Illumina HumanCoreExome array. The CHOP cohort consisted of 1229 cases from the USA and Norway, and 5512 paediatric controls, all recruited from within the CHOP Healthcare Network, and genotyped on the Illumina HumanHap550 or Human610-Quad arrays.^28^ Lastly, CLARITY^29^ consisted of 558 cases and 704 controls collected from the Royal Children’s Hospital and Monash Medical Centre in Melbourne. All controls and 406 cases were genotyped on the Illumina HumanCore array, with the remaining 152 cases genotyped on the Illumina HumanHap550 array.^16 17^

We applied consistent QC procedures across all the genotyped cohorts. The CLARITY cohort was genotyped in three batches and we performed QC separately in each. The QC was performed using plink1.9^31 32^ and included: removing non-autosomal SNPs, SNPs with minor allele frequency (MAF) <1%, SNPs and individuals with missingness >10%, and SNPs with deviations from Hardy-Weinberg equilibrium in controls (p<10^-3^). Additionally, using KING V.2.1.5,^33^ we identified and randomly removed one of two individuals with a second or higher degree relatedness within the cohorts. The resulting genotyped and QC cohorts are described in the online supplementary table S1.

10.1136/annrheumdis-2020-217421.supp1 Supplementary data

For genotype imputation of our QC cohorts, we used the Michigan Imputation Server^34^ with Minimac3 and the 2016 Haplotype Reference Consortium (HRC) as the reference panel. After imputation, we merged all the CLARITY batches into a single set. Then, for each consolidated cohort (UK, CHOP and CLARITY), we removed multi-allelic and duplicated SNPs, SNPs with imputation *r* ^2^ <0.5 and MAF <1%, SNPs deviating from the Hardy-Weinberg equilibrium in controls (p<10^-3^) and those with ambiguous strand (A/T or C/G alleles).

Next, we performed principal component analysis (PCA) using FlashPCA2^35 36^ over the filtered samples (online supplementary figures S1–S3). For each cohort, we selected the largest homogeneous subset of individuals based on visual inspection of the top five principal components (PCs) within each cohort. The UK cohort was kept in its entirety, while in CLARITY and CHOP, 168 and 3228 individuals were removed, respectively. Table 1 shows the final cohorts used in this work and online supplementary figures S4–5 show the PCA for these subsets. For the analysis, we used the n=5 545 761 genotyped and imputed SNPs which were available post-QC on all three cohorts.

**Table 1:** Cohort characteristics after imputation and quality control

	Total individuals	SNPs	Number of cases	Number of controls	Number of males	Number of females
UK	7505	6 029 891	2324	5181	3433	4072
CHOP	3513	6 338 131	559	2954	1671	1842
CLARITY	940	5 743 016	362	578	460	480

CHOP, Children’s Hospital of Philadelphia; CLARITY, ChiLdhood Arthritis Risk factor Identification sTudY; SNP, single nucleotide polymorphism.

### Development and validation of the genomic risk score

The UK cohort was used to train our models as it was the most homogeneous cohort with the largest case sample size (2324 cases, 5181 controls). To account for potential confounding by the case/control genotyping batch in the UK cohort, we used logistic regression of case/control status on sex and the first 10 genetic PCs. The PCs were computed over a subset of the SNPs of UK, excluding the HLA region as well as known or putative JIA risk loci^15^ (defined here as SNPs with p<10^-5^ and all SNPs within 1 Mb of the former). The residuals from the regression were then used as the phenotype for constructing the GRS.

To create the GRS we used SparSNP,^37^ which is an efficient implementation of a lasso-penalised linear model previously shown to outperform other methods when there are known to be strong effects within regions of complex linkage disequilibrium (LD), such as the major histocompatibility complex (MHC).^38^

SparSNP considers all post-QC SNPs in the training cohort for the construction of the model, but the final number of SNPs receiving a non-zero weight varies depending on the value of the penalties used, which were tuned via 10 repeats of 10-fold cross-validation. The optimal number of SNPs selected in the chosen model was decided based on the model with the highest average area under the receiver operating characteristic curve (AUC) across the replications (online supplementary material).

Once a model was chosen, we computed the GRSs for each of the test cohorts (CHOP and CLARITY). Assuming that the number of SNPs is m, then the GRS $g_{i}$ for a new individual with genotypes $x_{i}$ is

$$\begin{array}{l} g_{i}=\sum_{j=1}^{m}x_{ij}\hat{\beta_{j}} \end{array}$$

where *β_j_* are the SNP weights obtained from the model. Subsequently, in order to validate the GRS, we evaluated it using logistic regression in CHOP and CLARITY, adjusting for sex and first 10 genetic PCs of each test set.

## Results

### Training and external validation of the JIA genomic risk score

**Figure 2:**
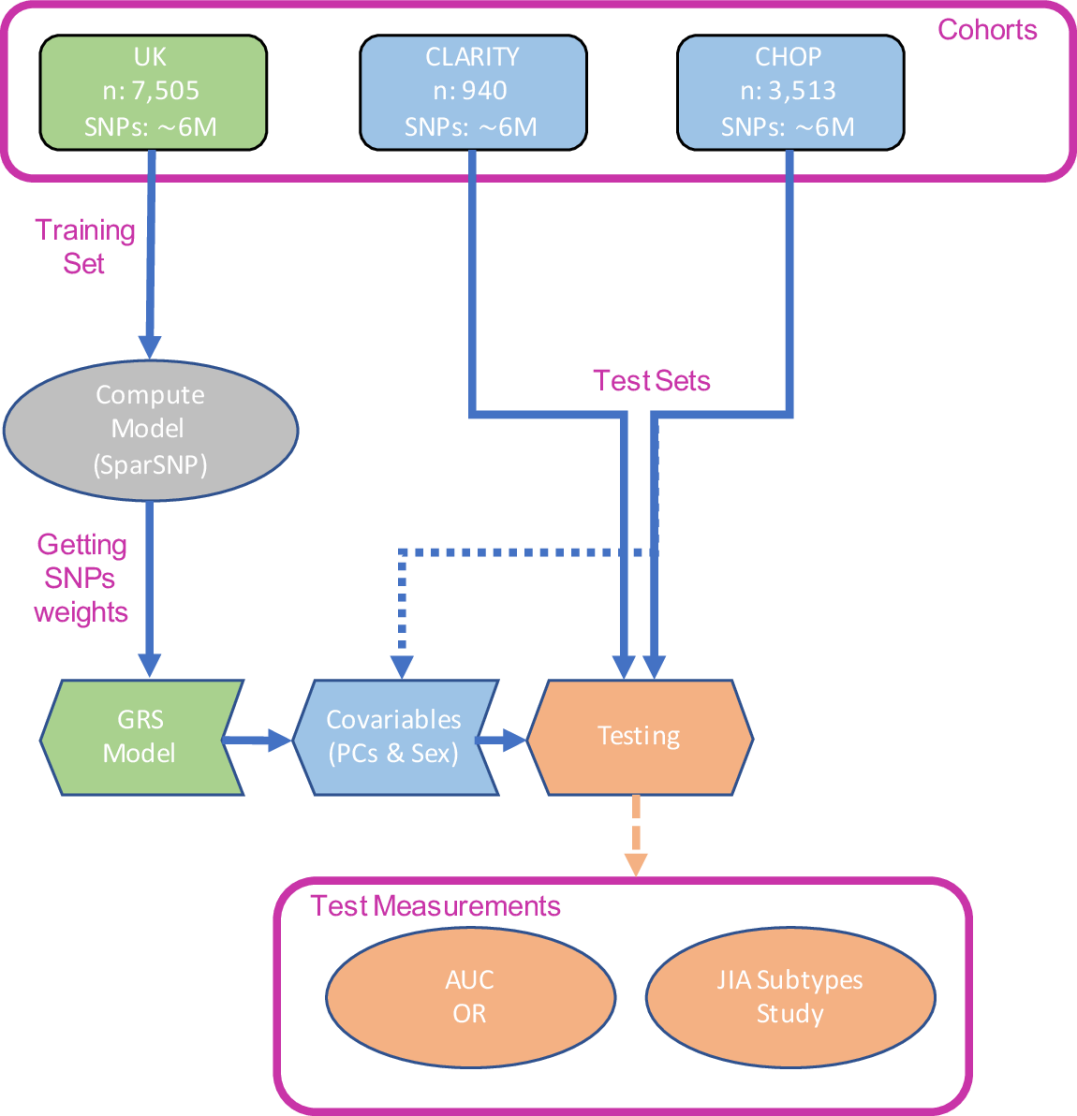
Outline of the study design followed in this work. AUC, area under the receiver operating characteristic curve; CHOP, Children’s Hospital of Philadelphia; CLARITY, ChiLdhood Arthritis Risk factor Identification sTudY; GRS, genomic risk score; JIA, juvenile idiopathic arthritis; PCs, principal components; SNP, single nucleotide polymorphism.

An overview of our study design is given in figure 2. We used 10×10-fold cross-validation to tune the penalised model and estimate the AUC in the UK cohort, achieving a maximum AUC=0.671 (95% CI 0.668 to 0.674) and selecting 26 SNPs in the model (online supplementary figure S6). The small number of SNPs selected is consistent with the strong HLA association of JIA (confirmed in a genome-wide association study (GWAS) of the UK cohort, online supplementary figure S7), and the way that SparSNP assigns weights to SNPs taking into account the correlation between them.^38^ The final model (SNP weights) was held fixed for external validation.

Before computing the GRSs, we estimated the SNP heritability of JIA in our cohorts using GCTA V.1.91.7^39 40^ and adjusted for first 10 PCs together with an assumed JIA population prevalence of 1/1000. The estimated SNP-heritabilities (on the liability scale) were *h* ^2^_SNP_=0.25 (SE 0.02) for the UK, *h* ^2^_SNP_=0.37 (SE 0.13) for CLARITY and *h* ^2^_SNP_=0.51 (SE 0.07) for CHOP. We corroborated these estimates using LDAK V.5.1^41^ (online supplementary table S2). Of the three estimates, the UK is likely the most reliable one due to its size and homogeneity, while CLARITY and CHOP were likely too small to derive reliable estimates. For comparison, we also derived the total narrow-sense heritability *h* ^2^ based on the sibling recurrence risk and population prevalence;^42^ a sibling recurrence risk of 11.6^18^ and a prevalence *K*=1/1000 are compatible with JIA narrow-sense heritability of 0.54 (for *K*=0.07/1000 and 4/1000, *h* ^2^=0.35 and 0.73, respectively).

We computed the GRS for CLARITY and CHOP, and evaluated the model in terms of AUC and ORs (table 2). Overall, the GRS showed highly consistent performance across both validation cohorts. The associations of the GRS with JIA status were unattenuated when adjusting for the top 10 genetic PCs and sex. Furthermore, we compared the GRS model with a model restricted to known SNPs previously shown to be associated with JIA.^15^ Using this restricted model, we achieved AUC of 0.614, 0.642 and 0.648 for the UK, CHOP and CLARITY, respectively, showing that the unrestricted GRS model was a stronger risk predictor.

**Table 2:** The predictive power of the GRS in the validation sets. Based on logistic regression on the test sets, optionally adjusted for sex and top 10 genetic PCs. Effect sizes are per SD of the GRS

	AUC (95% CI)	OR (95% CI)
CHOP		
Sex+PCs	0.677 (0.654 to 0.701)	
GRS	0.657 (0.631 to 0.683)	1.831 (1.685 to 1.991)
GRS+sex+PCs	0.735 (0.712 to 0.758)	1.838 (1.686 to 2.007)
CLARITY		
Sex+PCs	0.671 (0.636 to 0.706)	
GRS	0.671 (0.635 to 0.706)	2.008 (1.731 to 2.345)
GRS+sex+PCs	0.738 (0.705 to 0.770)	2.085 (1.773 to 2.471)

AUC, area under the receiver operating characteristic curve; CHOP, Children’s Hospital of Philadelphia; CLARITY, ChiLdhood Arthritis Risk factor Identification sTudY; GRS, genomic risk score; PCs, principal components.

Recent works have shown that a metaGRS approach can substantially improve genomic prediction of common diseases.^20 43^ Given the strong pleiotropy across autoimmune diseases, we hypothesised that it may be possible to extract more predictive signal from GRSs generated for other autoimmune diseases, via a meta-analytic strategy to construct a GRS for JIA that captures the totality of information from these GRSs into a single metaGRS for JIA. Thus, we computed a JIA metaGRS, based on a set of related autoimmune disease GWAS summary statistics, and compared with the GRS computed using lasso-penalised regression (online supplementary material). However, the JIA metaGRS’s performance was not significantly better than the original JIA GRS, thus subsequent analyses used the original model based on JIA alone.

### Subtype analysis

**Figure 3:**
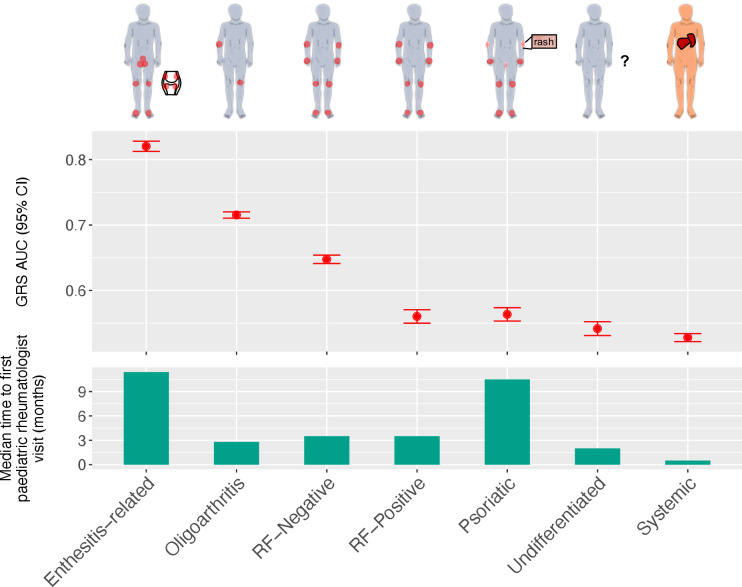
Cross-validated AUC achieved by training the seven JIA subtype specific models (top), and median time taken by an individual with JIA to be referred for first time to a paediatric rheumatologist visit (in months; bottom).^39^ AUC, area under the receiver operating characteristic curve; GRS, genomic risk score; JIA, juvenile idiopathic arthritis; RF-positive, rheumatoid-factor-positive polyarthritis; RF-negative, rheumatoid-factor-negative polyarthritis.

We extended our analysis to consider subtypes of JIA and construct subtype-specific GRSs thereof. The ILAR recognises seven subtypes of JIA: systemic arthritis, oligoarthritis, rheumatoid-factor-positive polyarthritis (RF-positive), rheumatoid-factor-negative polyarthritis (RF-negative), enthesitis-related arthritis (ERA), psoriatic arthritis and undifferentiated arthritis.^5^ Subtypes vary substantially in average age at onset, sex distribution, number of joints affected and clinical features (figure 3).^44^ Of particular interest clinically, is the time between onset of symptoms to visiting a paediatric rheumatologist, which has been estimated to vary from 11 months (range: 8 to 70 weeks) in the case of enthesitis-related JIA to 1 month (range: 2 to 36 weeks) for systemic JIA.^13 39^ The heterogeneity of JIA was reflected in our case data; all seven subtypes were present, although at frequencies ranging from common (~41% of UK cases were oligoarthritis JIA) to relatively rare (~2% of UK cases were undifferentiated JIA) (table 3).

**Table 3:** Characteristics of JIA subtypes across cohorts, including rate (%) of each subtype among cases of each cohort. Cases with no known subtype classification were excluded (n=29 from CLARITY and n=25 from the UK)

	UK (2324 cases)			CHOP (559 cases)			CLARITY (333 cases)		
	Rate (%)	Males	Females	Rate (%)	Males	Females	Rate (%)	Males	Females
Enthesitis-related	7.4	136	37	11.8	40	26	4.4	13	3
Oligoarthritis	41.1	299	657	36.3	39	164	43.9	42	117
RF-negative	23.8	144	408	24.2	34	101	20.7	23	52
RF-positive	5.5	13	115	5.2	1	28	3.0	1	10
Psoriatic	5.9	50	86	7.2	11	29	5.0	12	6
Undifferentiated	2.1	21	28	4.7	8	18	7.5	13	14
Systemic	13.2	142	164	10.6	24	36	7.5	12	15

CHOP, Children’s Hospital of Philadelphia; CLARITY, ChiLdhood Arthritis Risk factor Identification sTudY; JIA, juvenile idiopathic arthritis; RF-negative, rheumatoid-factor-negative polyarthritis; RF-positive, rheumatoid-factor-positive polyarthritis.

For each JIA subtype, we used the UK cohort to train subtype-specific GRSs, employing a similar approach as the JIA GRS above (**Methods**). Each subtype GRS was trained on the respective subtype’s cases and all controls, excluding other JIA subtypes cases from the training and validation cohorts. Online supplementary table S7 shows the number of SNPs selected by each subtype-specific model, and online supplementary figures S12–15 illustrate how the SNPs selected by different subtype-specific models correlated between them. In general, we observed that the SNPs selected by each model were distinct and few or none of them were in high LD with any SNP selected for another model, with the exception of the oligoarthritis and RF-negative models, which is consistent with previous studies.^44^ Once we computed the subtype-specific GRSs, we externally tested them in CHOP and CLARITY.

There was a high degree of variability in discrimination between subtype GRSs, with some subtypes displaying cross-validated AUCs greater than the JIA GRS and others not exhibiting significantly discrimination compared to random chance (AUC=0.5; figure 3). When compared with a previous estimate of the median time for a child with JIA to be referred to a paediatric rheumatologist visit,^39^ we found that the JIA subtype (ERA) with the longest time-to-referral (~11 months) also had the strongest subtype GRS (AUC=0.82 with 138 SNPs) and that the next strongest subtype GRS (AUC=0.71 with 25 SNPs) was for oligoarthritis JIA, which has a median time-to-referral of ~3 months. Since HLA-B27 antigen tests are a component of the ERA diagnosis criteria,^5 45^ we sought to evaluate whether *HLA-B27* genotypes were predictive of ERA status. The *HLA-B27* model from^46 47^ was used to classify each individual as *HLA-B27* positive or negative within each cohort (online supplementary material). The ERA GRS model's cross-validated AUC was higher than the AUC obtained using the *HLA-B27* model in the UK cohort (AUC=0.82 and AUC=0.79, respectively). This was consistent with external validations in CHOP (AUCs 0.698 and 0.678) and CLARITY (AUCs 0.838 and 0.803); however, the differences were not statistically significant using a DeLong test^48^ (online supplementary table S5). While the low frequency of ERA (CHOP: n=66, CLARITY: n=16) limited the test’s power, there was notable consistency of cross and external validation performance across all cohorts.

The weakest subtype GRSs were for the undifferentiated (AUC=0.542 with 1487 SNPs) and systemic (AUC=0.528 with 826 SNPs) subtypes. This was not unexpected as these subtypes are somewhat different to the other five subtypes. Children are diagnosed with the undifferentiated subtype when their symptoms do not fit within other subtypes, or meet the criteria for multiple subtypes. Systemic JIA is considered an autoinflammatory disease with little genetic overlap with the other JIA subtypes.^49^ However, it has been shown that systemic JIA has strong genetic signals from the HLA class II molecule encoded by HLA-DRB1*11, confirming the role of the adaptive immune system.^50^ To test this, we statistically imputed HLA-DRB1*11 risk alleles using HIBAG V.1.20^51 52^ in the three cohorts, tested the predictive power of these genotypes, and compared them with our systemic JIA GRS in CHOP and CLARITY (online supplementary table S6). In the UK cohort, the HLA-DRB1*11 model was associated with systemic JIA (OR=1.31, 95% CI 1.19 to 1.43) and had a higher AUC (0.563) than in the cross-validated UK systemic JIA GRS model. However, its performance on the external cohorts was inferior to using the systemic JIA GRS, as the HLA-DRB1*11 frequencies were the same in systemic JIA cases and the controls (CHOP:~18%; CLARITY:~25%), unlike the UK cohort (~25% in cases and ~12% in controls). Given the heterogeneity across cohorts, interpreting these results is challenging particularly because systemic JIA is relatively rare, and *HLA-DRB1* alleles are often less accurately imputed than other alleles.^51 53^

In general, external validation of the subtype-specific GRSs in CLARITY showed highly consistent AUC estimates with cross-validation performance in the UK, while in the CHOP cohort there was somewhat less consistent external validation than CLARITY (table 4).

**Table 4:** External validation of the subtype-specific GRSs in CLARITY and CHOP. Based on logistic regression on the test sets, optionally adjusted for sex and top 10 genetic principal components. Effect sizes are per SD of the GRS

	AUC (95% CI)		OR (95% CI)	
	CHOP	CLARITY	CHOP	CLARITY
Enthesitis-related				
GRS	0.70 (0.63 to 0.77)	0.84 (0.71 to 0.97)	1.84 (1.60 to 2.17)	2.99 (2.11 to 4.54)
GRS+sex+PCs	0.75 (0.68 to 0.82)	0.93 (0.86 0.99)	1.86 (1.61 to 2.14)	3.09 (2.07 to 5.04)
Oligoarthritis				
GRS	0.77 (0.73 to 0.80)	0.74 (0.70 to 0.79)	1.93 (1.76 to 2.11)	2.24 (1.88 to 2.71)
GRS+sex+PCs	0.80 (0.77 to 0.84)	0.79 (0.76 to 0.83)	1.93 (1.75 to 2.13)	2.19 (1.81 to 2.71)
RF-negative				
GRS	0.64 (0.59 to 0.69)	0.66 (0.59 to 0.73)	1.48 (1.33 to 1.64)	1.69 (1.42 to 2.02)
GRS+sex+PCs	0.76 (0.72 to 0.80)	0.74 (0.68 to 0.80)	1.51 (1.35 to 1.68)	1.71 (1.42 to 2.07)
RF-positive				
GRS	0.57 (0.47 to 0.67)	0.59 (0.40 to 0.78)	0.73 (0.44 to 1.11)	1.42 (0.85 to 2.17)
GRS+sex+PCs	0.79 (0.73 to 0.86)	0.97 (0.94 to 0.99)	0.74 (0.44 to 1.13)	1.27 (0.60 to 2.52)
Psoriatic				
GRS	0.56 (0.47 to 0.65)	0.58 (0.44 to 0.73)	0.77 (0.52 to 1.08)	1.33 (0.87 to 1.91)
GRS+sex+PCs	0.70 (0.62 to 0.78)	0.76 (0.66 to 0.85)	0.77 (0.52 to 1.08)	1.32 (0.85 to 1.96)
Undifferentiated				
GRS	0.48 (0.35 to 0.61)	0.52 (0.42 to 0.62)	0.89 (0.60 to 1.31)	0.89 (0.59 to 1.31)
GRS+sex+PCs	0.69 (0.58 to 0.80)	0.75 (0.66 to 0.83)	0.90 (0.60 to 1.33)	0.82 (0.51 to 1.28)
Systemic				
GRS	0.50 (0.43 to 0.58)	0.52 (0.41 to 0.62)	1.01 (0.78 to 1.30)	1.07 (0.73 to 1.56)
GRS+sex+PCs	0.69 (0.62 to 0.76)	0.75 (0.66 to 0.84)	1.01 (0.78 to 1.30)	1.13 (0.73 to 1.72)

AUC, area under the receiver operating characteristic curve; CHOP, Children’s Hospital of Philadelphia; CLARITY, ChiLdhood Arthritis Risk factor Identification sTudY; GRS, genomic risk score; PCs, principal components.

## Discussion

The accurate and timely diagnosis of JIA is a currently unmet clinical need. In this study, we aimed to address the paucity of molecular tools to aid the entirely clinical diagnosis of JIA, by leveraging the wealth of human genomic data gathered over the last decade, and developing a series of GRSs for JIA. We have shown that genomic machine learning can yield predictive GRSs for JIA as a composite diagnosis as well as subtype-specific GRSs, including the most common clinically reported subtype (oligoarthritic JIA),^54^ as well as enthesitis-related JIA, which can present with non-specific pains initially and is therefore more difficult to diagnose clinically. Given the cost effectiveness of a genotyping array and the time-invariant properties of germline DNA, these JIA GRSs hold promise for rapid clinical translation as means of diagnosis and risk stratification. At-risk children can be non-invasively stratified as high-risk much earlier in the diagnostic pathway, and children with low risk non-inflammatory disease can be appropriately triaged and managed earlier. To facilitate translation and clinical uptake, we have made the genetic variants and weights of our JIA GRSs publicly available via the Polygenic Score (PGS) Catalog (http://www.pgscatalog.org/pgs/PGS000114/).

A strength of this study is that the JIA GRS was developed on a UK data set and externally validated in two independent studies in Australia and the USA, indicating the robustness of the score. Despite having used the largest JIA cohorts available currently, the scores developed here only partially explained the genetic variability in JIA. Future improvements in predictive power will likely come with larger cohorts, particularly for less-common subtypes. In the case of the ERA subtype, we found that the GRS AUC was greater than the HLA haplotype in the UK, Australian and US-based cohorts. However, we caution that larger cohorts will be necessary for powerful statistical testing and assessment of clinical utility of GRS as compared with HLA typing for both ERA and systemic JIA. Furthermore, given the genetic heterogeneity of JIA subtypes, our study demonstrates that adding genomics to the ILAR classification has potential to increase the efficiency of classification, and may in turn inform the refinement or even redefinition of JIA subtype classification. However, we also caution that a limitation of the current study is that the participants in our cohorts were of European descent and we were unable to assess the performance of the JIA GRS in individuals of non-European ancestries,^55^ which will be crucial for wide-spread clinical deployment of such scores.

In both primary and tertiary healthcare settings, it is often challenging to recognise and diagnose JIA in children, as there are many non-inflammatory conditions that are common to children that present with musculoskeletal pain mimicking JIA. Difficulty in discriminating between these cases causes delays in accessing vital care, due to the multitude of investigations and assessments that need to be done first. Moreover, accessing paediatric rheumatology specialist services is difficult, as waiting lists are usually lengthy and access to care is problematic due to workforce shortages worldwide.^56^ Currently, there are no sensitive or specific tests available to assist clinicians in making the diagnosis of JIA. In a well-resourced setting, a clinician will typically use a combination of history, examination, blood tests such as inflammatory markers, rheumatoid factor (positive in 7%), *HLA-B27* (varying on subtype but present in between 10% to 74% cases); or medical imaging, such as X-ray, ultrasound or MRI. The potential burden of such repeated testing on children and their families can be high, both in a socioeconomic sense and psychological sense. Therefore, in the hands of a primary care doctor a diagnostic algorithm based on a JIA-GRS may provide a more timely, accessible and reliable means of assessing children with musculoskeletal symptoms who may be JIA cases, thus enabling appropriate triage and referral, facilitating early access to appropriate care, and reducing the pain, complications of the disease and poor long-term health outcomes, due to delayed diagnosis and treatment.
