# Supplementary material for: Genomic risk scores for juvenile idiopathic arthritis and its subtypes
Source: Ann Rheum Dis. 2020 Sep 4;79(12):1572–9. doi: 10.1136/annrheumdis-2020-217421 (PMC7677485; doi:10.1136/annrheumdis-2020-217421)
Supplement: Supplementary data [file annrheumdis-2020-217421supp001.pdf]

Genomic risk scores for juvenile idiopathic arthritis and its subtypes

Rodrigo Cánovas, Joanna Cobb, Marta Brozynska, John Bowes, Yun R. Li, Samantha L Smith, Hakon Hakonarson, Wendy Thomson, Justine Ellis, Gad Abraham, Jane Munro, Michael Inouye

Supplementary Material

Creating the genomic risk score model

We constructed the models using as phenotype the residuals from the logistic regression of case/control status on sex and the first 10 genetic PCs, on the UK cohort. The PCs were computed over a subset of the SNPs of UK, excluding the HLA region as well as known or putative JIA risk loci[1] (defined here as SNPs with $P<10^{-5}$ and all SNPs within 1 Mb of the former).

To train the genomic risk scores (GRS) models for this work, we used the UK cohort and SparSNP[2], which is an efficient implementation of a lasso-penalised linear model. They were induced by minimising the L1-penalised squared loss function (lasso) over *N* individuals and *m* SNPs,

$$L(\hat{\beta}_0, \hat{\boldsymbol{\beta}}) = \frac{1}{2N}\sum_{i=1}^{N}\left(y_i - \hat{\beta}_0 - \boldsymbol{x}_i^T\hat{\boldsymbol{\beta}}\right)^2 + \lambda\sum_{j=1}^{m}\left|\hat{\beta}_j\right|, \quad (1)$$

where x_i is the m-vector of genotypes for the ith sample in allele-dosage coding $\{0,1,2\}$, y_i is the phenotype, $\hat{\boldsymbol{\beta}}$ is the m-vector of weights, $\hat{\beta}_0$ is the intercept, and λ is the L1 penalty. All SNPs in the selected training dataset are considered in the model but typically only a small number of SNPs receive a non-zero weight. The number of SNPs with non-zero weight varies depending on the value of the L1 penalty (higher penalties lead to fewer SNPs), which was tuned via 10x10-fold cross-validation. We also explored adding an L2 penalty to the model (elastic-net); however, no significant improvement was found.

MetaGRS

We explored an alternative approach to enhance the predictive power of our current JIA model. Given the strong pleiotropy across autoimmune diseases, we hypothesised that it may be possible to extract more predictive signal from GRSs generated for other autoimmune diseases via a metaGRS approach[3,4], particularly given the limited sample sizes available for JIA GWAS.

Figure S8 illustrates the pipeline followed to compute the metaGRS model. **Table S3** shows the 16 autoimmune disease summary statistic (GWAS) sets that were considered. We generated GRSs by LD-clumping (plink --clump) each summary statistic using the CLARITY cohort. **Figure S9** shows the correlations between the GRSs including our JIA GRS model. Next, using CLARITY as a training set, we applied elastic-net regression in ten-fold cross-validation (using the *glmnet* R package[5]) to combine all the GRSs into a single weighted 'meta' GRS. **Figure S10** shows the model performance (binomial deviance) across a range of values of the elastic net penalty, in cross-validation on the training set, where we selected the

Cánovas R, *et al. Ann Rheum Dis* 2020; 79:1572–1579. doi: 10.1136/annrheumdis-2020-217421

model with the lowest binomial deviance (the parameter s="lambda.min" in the *glmnet* package). Finally, the weights for this model were used to construct the JIA metaGRS model.

Figure S11 compares the effect size (odds ratio, OR) of each of the GRSs individually (logistic regression) and their effect size as part of the JIA metaGRS (lasso logistic regression), in CLARITY. Finally, **Table S4** presents the OR and AUC obtained by our JIA GRS and metaGRS model GRSs. The results showed improvement of >1% in AUC in compare with JIA GRS, however the uncertainty in estimates was large due to limited sample sizes.

Enthesitis-related JIA *HLA-B27* and Systemic JIA *HLA-DRB1*11* models

The human leukocyte antigen B27 gene (*HLA-B27*) has a strong association with the enthesitis-related arthritis JIA subtype[6–10]. In order to predict enthesitis-related arthritis based on the presence or absent of the *HLA-B27* gene in each cohort, we created a model by using the most commonly tested tag SNPs for *HLA-B2*7 (rs13202464, rs116488202 and rs4349859)[6,7] to classify each individual as *HLA-B27* positive or negative. Then we ran a logistic regression model between the case/control status and the *HLA-B27* presence in each cohort. **Table S5** shows the performance of the computed ERA JIA GRS and the *HLA-B27* model in the external ERA subset validation datasets, CHOP and CLARITY.

Similarly, *HLA-DRB1*11* is strongly associated with systemic JIA [11]. We imputed the *HLA-DRB1*11* alleles in each cohort using the HIBAG v1.20 tool [12] and ran logistic regression on the case/control status and the imputed *HLA-DRB1*11* alleles in the dataset. **Table S6** shows the performance of the computed systemic JIA GRS and the *HLA-DRB1*11* model in the external systemic subset validation datasets, CHOP and CLARITY.

Cánovas R, *et al. Ann Rheum Dis* 2020; 79:1572–1579. doi: 10.1136/annrheumdis-2020-217421

Supplementary Tables

Table S1: Genotyped and quality-controlled cohort characteristics before imputation and removal of outliers. The final sets used to create and validate the model in this work can be found on **Table 1** in the main paper.

	Total individuals	SNPs	Number of cases	Number of controls
**UK**	7,505	144,964	2,324	5,181
**CHOP**	6,741	489,672	1,229	5,512
**CLARITY**				
**2010**	152	490,629	152	0
**2016**	838	246,814	247	591
**2017**	118	243,662	5	13

Table S2: Estimated SNP heritability (standard error) computed using GCTA 1.91.7[13] and LDAK v5.1[14] over each of the cohorts subsets used in this work. The estimated SNP heritability h^2_{SNP} was adjusted for 10 principal components as fixed effects, and a population prevalence of K=1/1000 was assumed. The results are on liability scale. Of these estimates, the UK is likely the most reliable one due to its size and homogeneity, while CLARITY and CHOP are likely too small to derive reliable estimates using the GCTA and LDAK tool.

	GCTA h^2_{SNP} (s.e.)	LDAK h^2_{SNP} (s.e.)
**UK**	0.25 (0.02)	0.27 (0.02)
**CHOP**	0.51 (0.07)	0.59 (0.07)
**CLARITY**	0.37 (0.13)	0.37 (0.00)

Cánovas R, *et al. Ann Rheum Dis* 2020; 79:1572–1579. doi: 10.1136/annrheumdis-2020-217421

Table S3: List of external summary statistics (GWAS) used to compute the JIA MetaGRS. and the final number of SNPs with non-zero weight from each GRS.

GRS label	Trait name	Number of SNPs after clumping	Study reference
SLE	Systemic lupus erythematosus	1,022	[15]
MS	Multiple Sclerosis	546	[16]
MS (ic)	Multiple Sclerosis	964	[17]
PSO	Psoriasis	615	[18]
NAR	Narcolepsy	44	[19]
CEL	Celiac	1,141	[20]
T1D (cc)	Type 1 Diabetes	380	[21]
T1D (meta)	Type 1 Diabetes	457	[21]
RA (Okada)	Rheumatoid Arthritis	1,872	[22]
RA (Stahl)	Rheumatoid Arthritis	816	[23]
RA (Eyre)	Rheumatoid Arthritis	658	[24]
PBC (Liu)	Primary Biliary Cirrhosis	449	[25]
PBC (Cordell)	Primary Biliary Cirrhosis	524	[26]
AS	Ankylosing Spondylitis	378	[6]
UC	Ulcerative Colitis	827	[27]
JIA (Hinks)	Juvenile Idiopathic Arthritis	256	[1]

Table S4: Performance of the JIA GRS and metaGRS in external validation on the CHOP cohort. Based on logistic regression, optionally adjusting for sex and top 10 genetic principal components (PCs).

	AUC (95% CI)	OR (95% CI)
**CHOP**		
Sex + PCs	0.677 (0.654–0.701)	--
GRS	0.657 (0.631–0.683)	1.831 (1.685–1.991)
MetaGRS	0.684 (0.659–0.709)	2.051 (1.870–2.252)
GRS + Sex + PCs	0.735 (0.712–0.758)	1.838 (1.686–2.007)
MetaGRS + Sex + PCs	0.748 (0.725–0.771)	2.042 (1.857–2.250)

Cánovas R, *et al. Ann Rheum Dis* 2020; 79:1572–1579. doi: 10.1136/annrheumdis-2020-217421

Table S5: Performance of the ERA GRS and the *HLA-B27* model (AUC and odds ratios (OR)) in the ERA subset of the CHOP (66 cases) and CLARITY (16 cases) cohorts. Based on logistic regression, optionally adjusting for sex and top 10 genetic principal components. Effect sizes are per standard deviation of the GRS and *HLA-B27* status respectively. Additionally, the table presents the results obtained from a DeLong test[28] of the difference in AUC between the ERA GRS and the *HLA-B27* model.

	AUC (95% CI)	OR (95% CI)	DeLong Test Z-statistic	DeLong Test p-value
**CHOP**				
GRS	0.698 (0.626–0.770)	1.840 (1.598–2.116)	0.900	0.368
HLA-B27	0.678 (0.617–0.739)	1.897 (1.588–2.266)	-	-
GRS + Sex + PCs	0.751 (0.684–0.818)	1.857 (1.608–2.140)	0.210	0.834
HLA-B27 + Sex + PCs	0.748 (0.682–0.815)	1.913 (1.598–2.291)	-	-
**CLARITY**				
GRS	0.838 (0.705–0.972)	2.989 (2.113–4.535)	0.985	0.325
HLA-B27	0.803 (0.693–0.914)	2.881 (1.938–4.630)	-	-
GRS + Sex + PCs	0.930 (0.860–0.999)	3.095 (2.074–5.042)	1.390	0.165
HLA-B27 + Sex + PCs	0.923 (0.854–0.993)	3.562 (2.202–6.438)	-	-

Table S6: Performance of the systemic JIA GRS and the *HLA-DRB1*11* model (AUC and odds ratio (OR)) in the systemic subset of the CHOP (60 cases) and CLARITY (27 cases) datasets. Based on logistic regression, optionally adjusting for sex and top 10 genetic principal components (PCs). Effect sizes are per standard deviation of the GRS and *HLA-DRB1*11* status respectively. Additionally, the table presents the results obtained from a DeLong test[28] of the difference in AUC between the systemic JIA GRS and the *HLA-DRB1*11* model.

	AUC (95% CI)	OR (95% CI)	DeLong Test Z-statistic	DeLong Test p-value
**CHOP**				
GRS	0.502 (0.427–0.579)	1.008 (0.778–1.297)	0.114	0.909
*HLA-DRB1*11*	0.497 (0.448–0.546)	0.994 (0.750–1.261)	-	-
GRS + Sex + PCs	0.689 (0.624–0.755)	1.009 (0.776–1.302)	-0.770	0.442
*HLA-DRB1*11* + Sex + PCs	0.690 (0.624–0.755)	0.995 (0.750–1.266)	-	-
**CLARITY**				
GRS	0.518 (0.414–0.621)	1.072 (0.725–1.556)	0.277	0.782
*HLA-DRB1*11*	0.500 (0.416–0.583)	0.958 (0.613–1.380)	-	-
GRS + Sex + PCs	0.750 (0.657–0.842)	1.130 (0.730–1.723)	-0.052	0.958
*HLA-DRB1*11* + Sex + PCs	0.750 (0.654–0.847)	1.160 (0.708–1.799)	-	-

Cánovas R, *et al. Ann Rheum Dis* 2020; 79:1572–1579. doi: 10.1136/annrheumdis-2020-217421

Table S7: Number of non-zero weighted SNPs used by each subtype-specific JIA model and number of SNPs shared between different model. The values on the diagonal indicate the number of SNPs with non-zero weigh within each subtype-specific model, and the rest of the values indicate the number of SNPs shared between different models.

	Enthesitis-related	Oligoarthritis	RF-Negative	RF-Positive	Psoriatic	Undifferentiated	Systemic
**Enthesitis-related**	**138**						
**Oligoarthritis**	0	**21**					
**RF-Negative**	0	6	**12**				
**RF-Positive**	2	0	0	**83**			
**Psoriatic**	3	0	0	1	**82**		
**Undifferentiated**	2	0	0	2	0	**1,487**	
**Systemic**	5	0	0	4	2	8	**826**

Cánovas R, *et al. Ann Rheum Dis* 2020; 79:1572–1579. doi: 10.1136/annrheumdis-2020-217421

Supplementary Figures

Figure S1: Top five principal components for the final UK imputed and quality-controlled cohort used to train all models in this work.

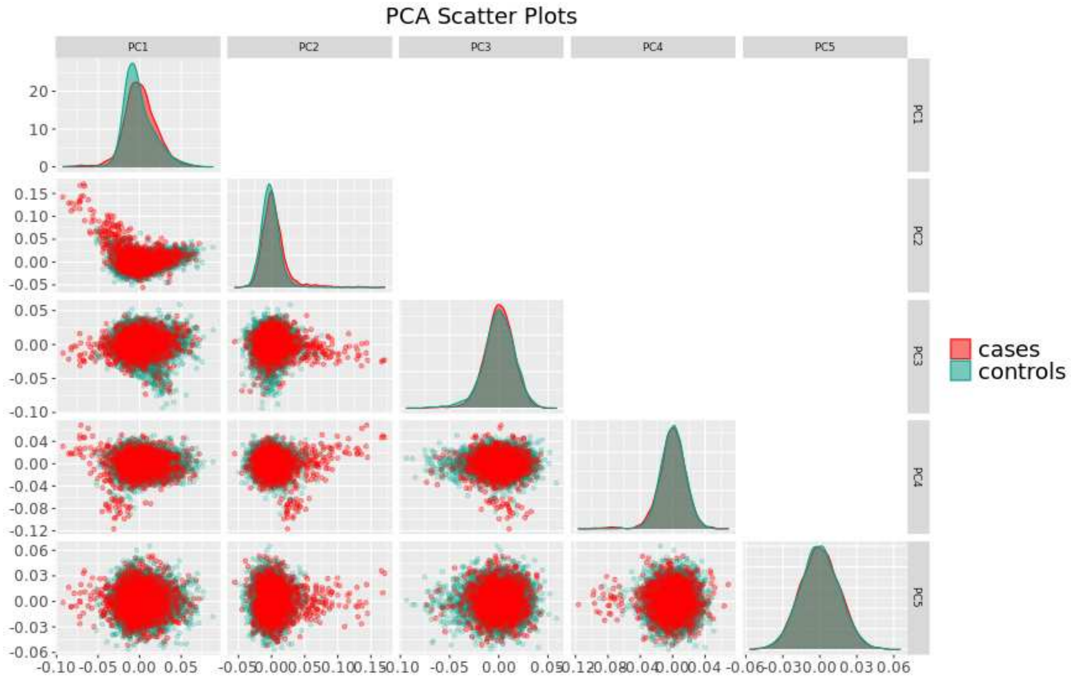

Figure S2: Top five principal components for the CHOP imputed and quality controlled cohort. Additionally, the figure shows the thresholds used for filtering outliers ($0.0 \leq PC1 \leq 0.05, -0.01 \leq PC2 \leq 0.02, -0.05 \leq PC3 \leq 0.06, -0.025 \leq PC4 \leq 0.055, -0.025 \leq PC5 \leq 0.042$).

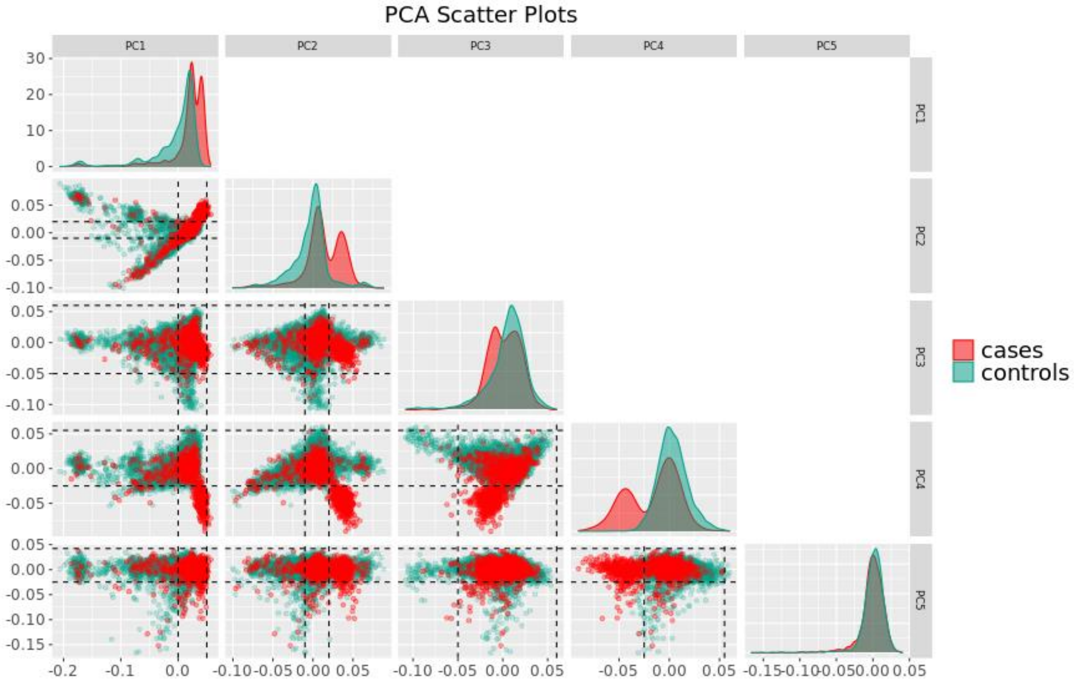

Cánovas R, *et al. Ann Rheum Dis* 2020; 79:1572–1579. doi: 10.1136/annrheumdis-2020-217421

Figure S3: Top five genetic principal components for the CLARITY imputed and quality controlled cohort. Additionally, the figure shows the thresholds used for filtering outliers ($0.07 \leq PC1 \leq 0.05, -0.06 \leq PC2 \leq 0.15, -0.15 \leq PC3 \leq 0.07, -0.06 \leq PC4 \leq 0.05, -0.06 \leq PC5 \leq 0.1$).

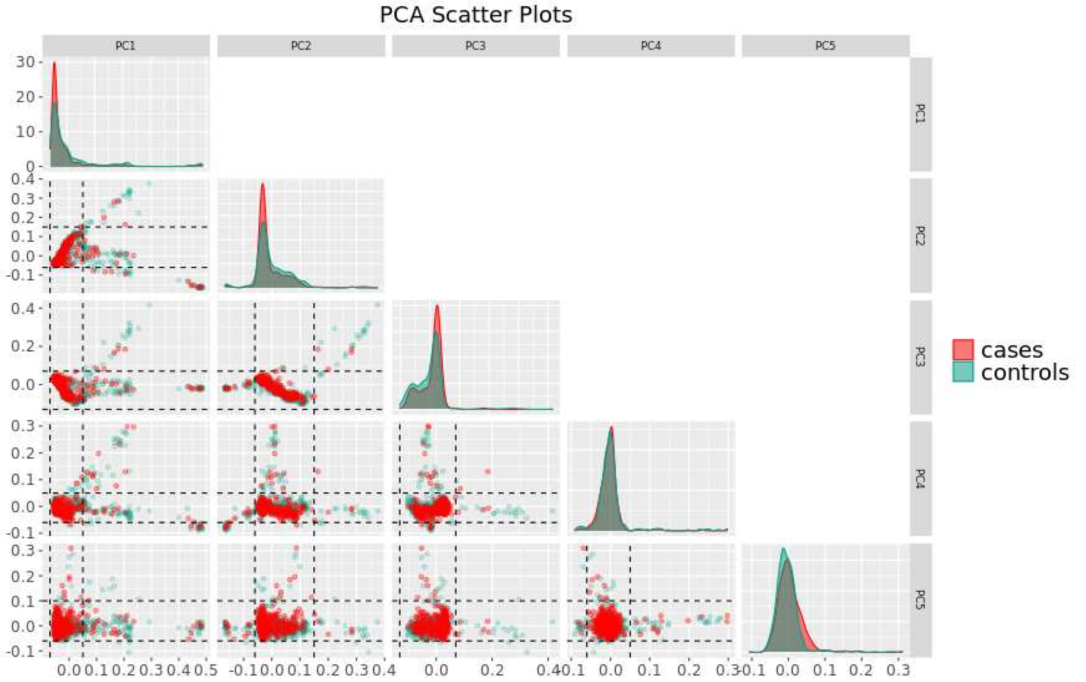

Figure S4: Top five genetic principal components for the CHOP subset used as validation cohort in this work. The subset of individuals were selected by excluding outliers from the original data based on visual inspection of the top five genetic principal components (**Figure S2**).

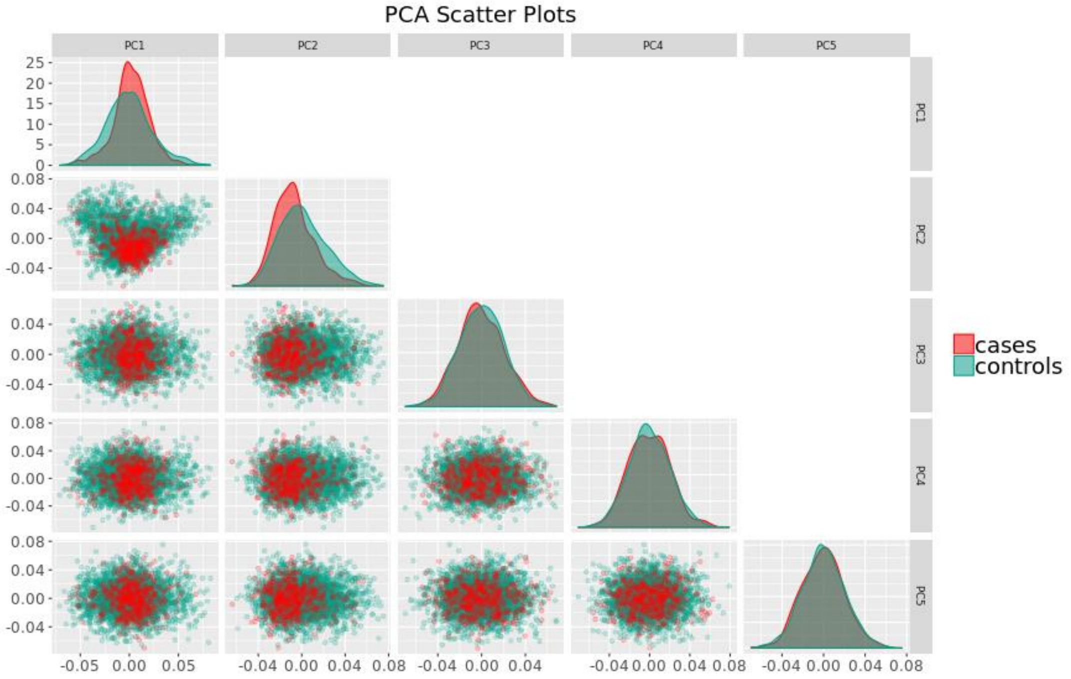

Cánovas R, *et al. Ann Rheum Dis* 2020; 79:1572–1579. doi: 10.1136/annrheumdis-2020-217421

Figure S5: Top five genetic principal components for the CLARITY subset used as validation cohort in this work. The subset of individuals were selected by excluding outliers from the original data based on visual inspection of the top five genetic principal components (**Figure S3)**.

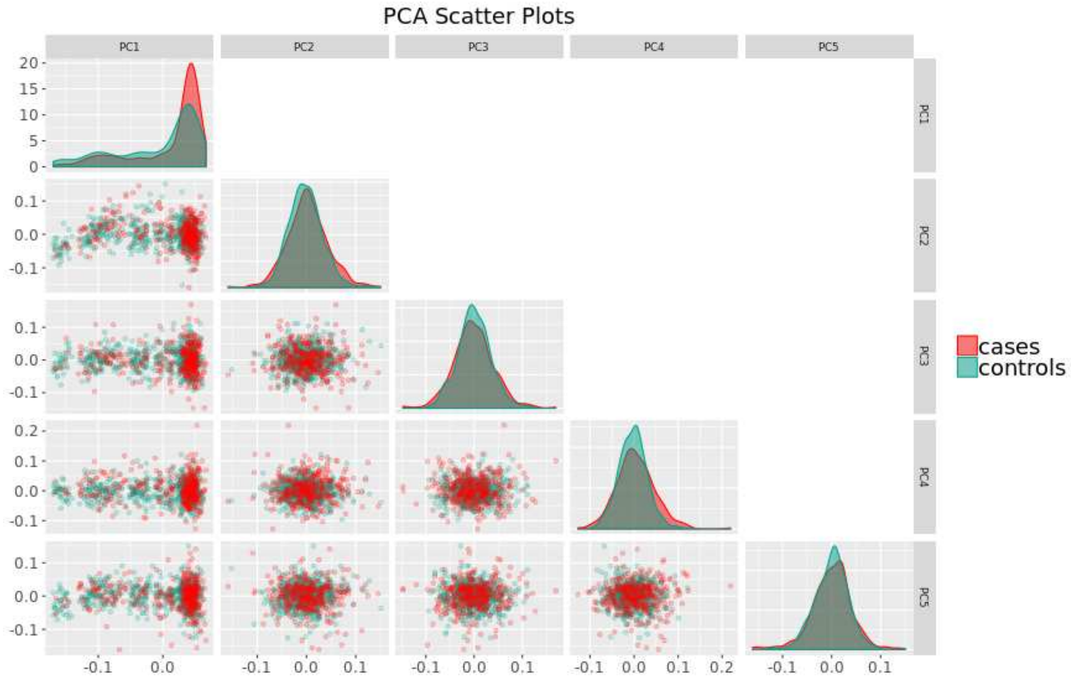

Figure S6: Cross-validation results within the UK cohort. The results are from 10x10 cross-validated AUC (LOESS-smoothed) as a function of the number of SNPs assigned a non-zero weight in the model. The best model was selected at 26 SNPs with AUC=0.671 (95% CI 0.668–0.674).

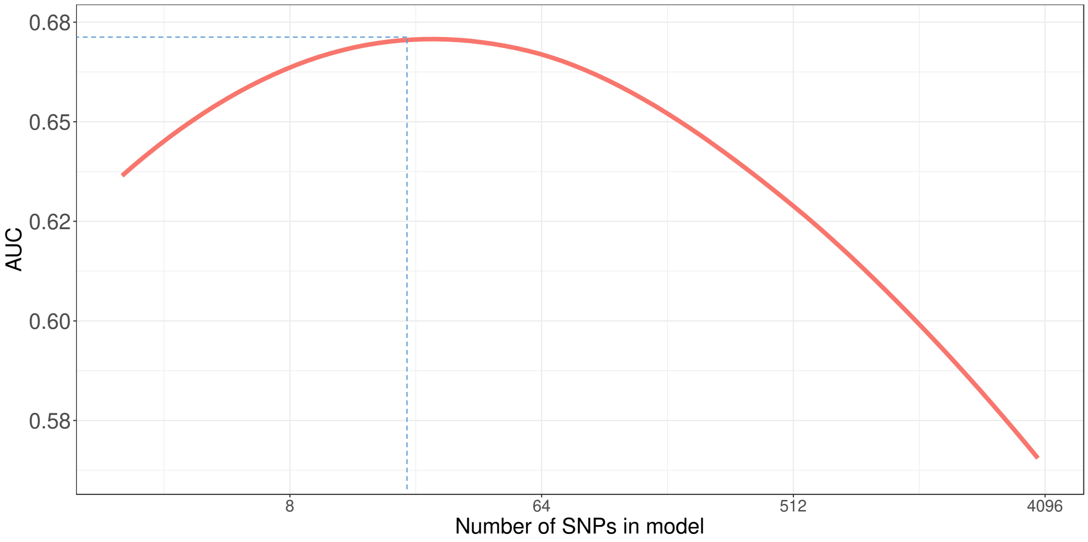

Cánovas R, *et al. Ann Rheum Dis* 2020; 79:1572–1579. doi: 10.1136/annrheumdis-2020-217421

Figure S7: A) Genome-wide association study (GWAS) of the UK cohort adjusted by its top ten genetic principal components. The 26 green highlighted points correspond to the SNPs with non-zero weight selected by the GRS model. B) Weights associated for each of the 26 SNPs selected for the model.

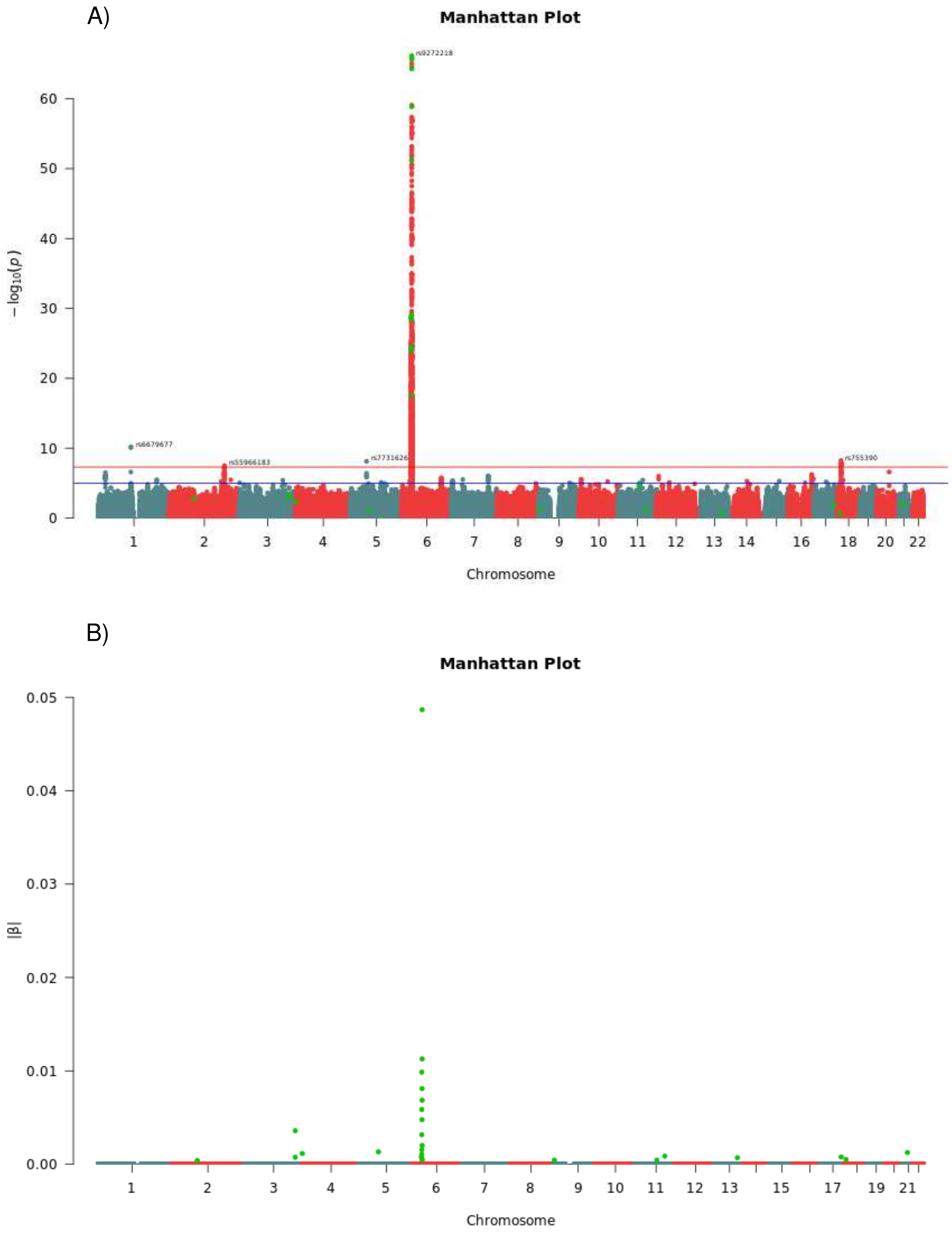

Cánovas R, *et al*. *Ann Rheum Dis* 2020; 79:1572–1579. doi: 10.1136/annrheumdis-2020-217421

Figure S8: Workflow followed to develop the JIA MetaGRS.

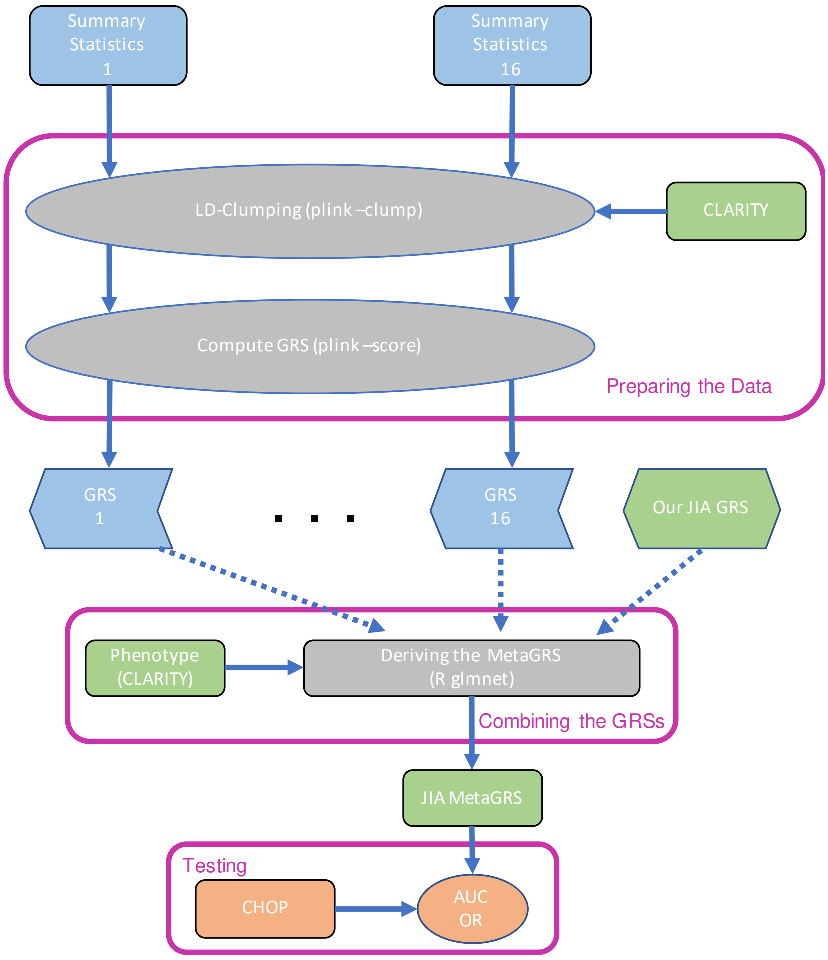

Cánovas R, *et al*. *Ann Rheum Dis* 2020; 79:1572–1579. doi: 10.1136/annrheumdis-2020-217421

Figure S9: Correlation matrix between the JIA GRS model and all the external GRSs generated (refer to **Table S3**) to create the JIA MetaGRS model. All the GRSs were computed using the CLARITY cohort.

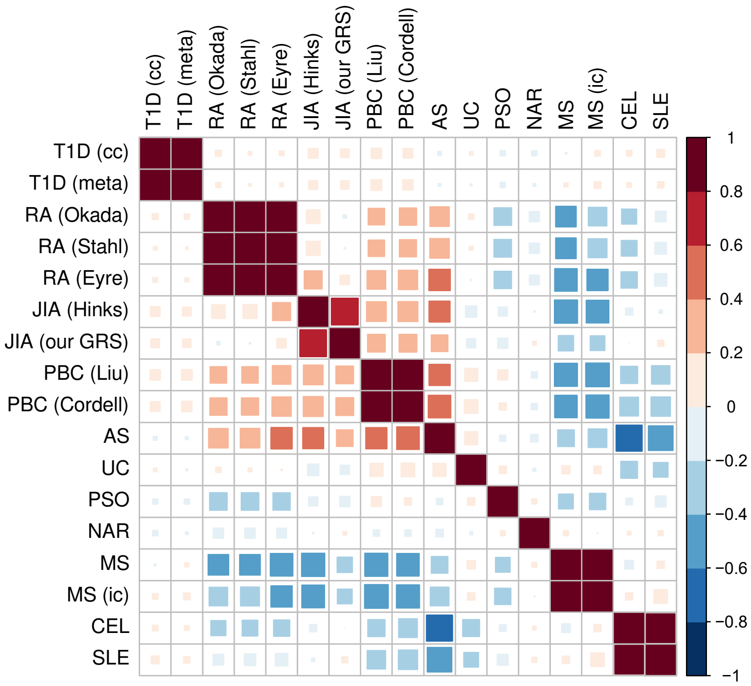

Figure S10: 10-fold cross-validated binomial deviance from elastic-net regression on JIA phenotype using the JIA GRS model in combination with the 16 autoimmune GRSs (refer to **Table S3**).

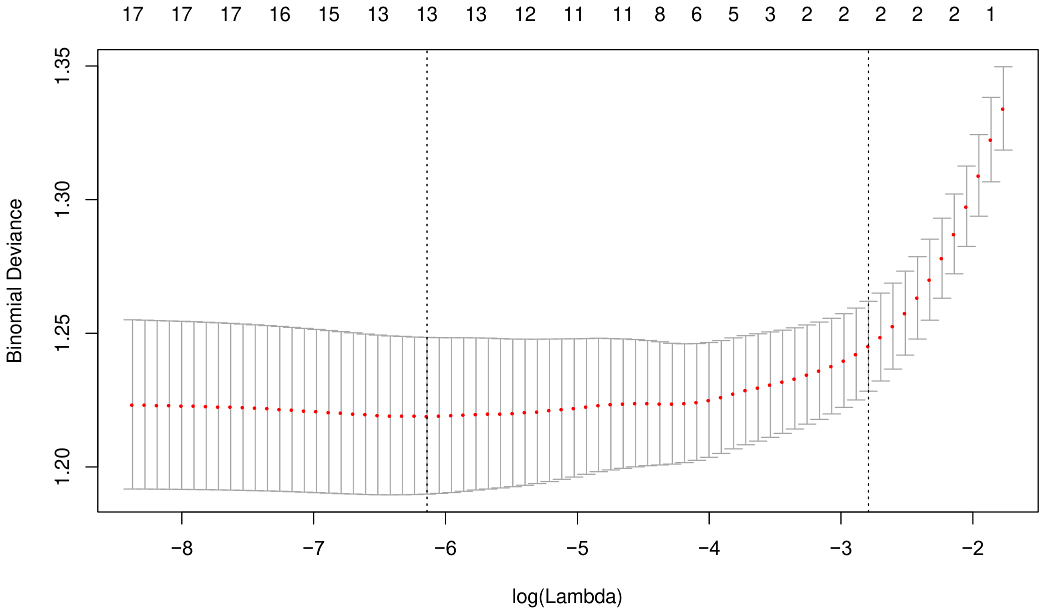

Cánovas R, *et al. Ann Rheum Dis* 2020; 79:1572–1579. doi: 10.1136/annrheumdis-2020-217421

Figure S11: Comparison of the effect size (95% CI) of each of the GRSs (refer to **Table S3**) in (i) logistic regression (considering each GRS individually) vs (ii) the conditional effect size in elastic-net logistic regression (used to create the metaGRS), in the CLARITY cohort. Confidence intervals are not available for the odds ratios estimated via elastic-net.

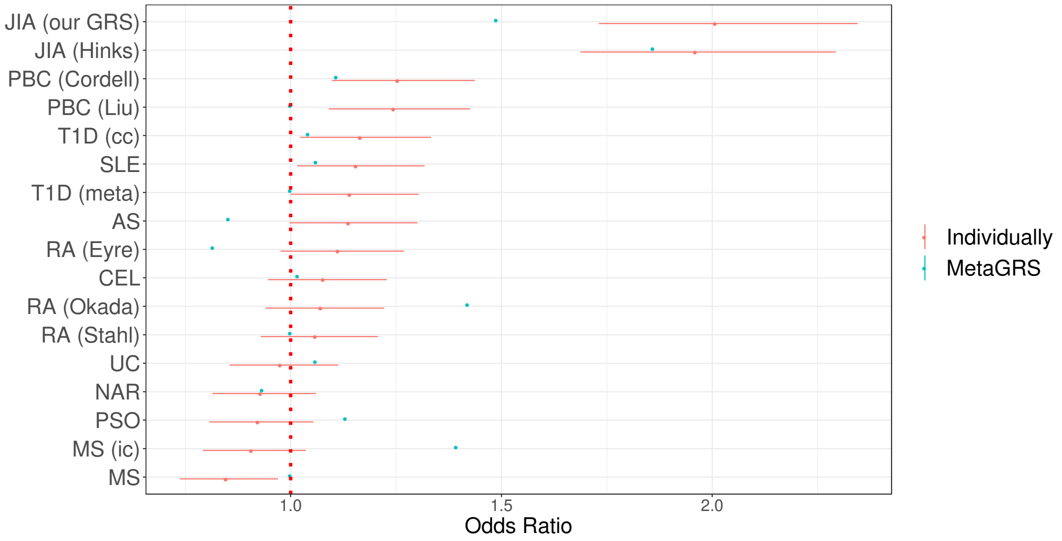

Cánovas R, *et al. Ann Rheum Dis* 2020; 79:1572–1579. doi: 10.1136/annrheumdis-2020-217421

Figure S12: r^2 between the SNPs selected by each subtype-specific JIA model. Each column and row in the matrices represent one SNP in the model and SNPs are ordered by chromosome and position.

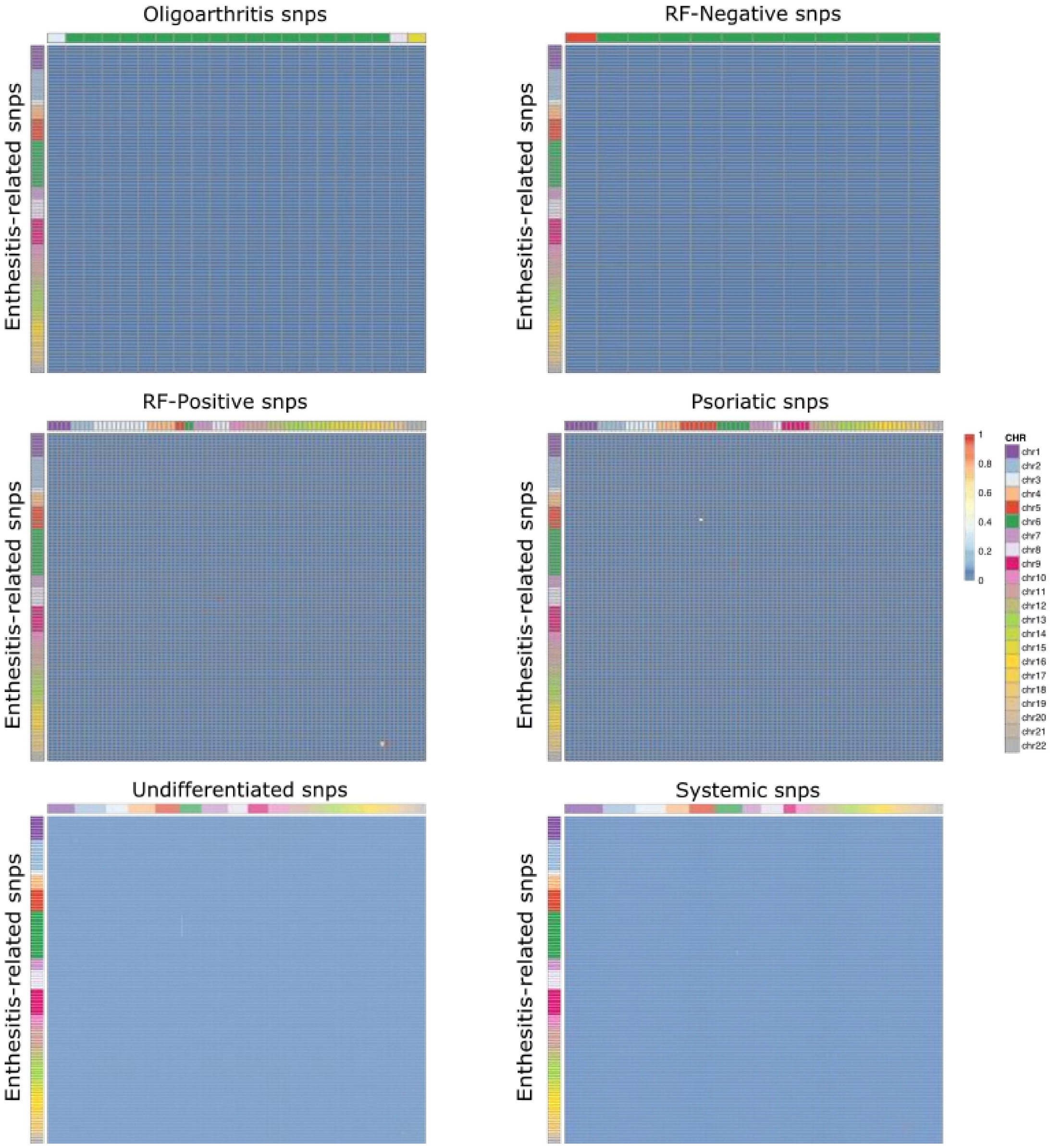

Figure S13: r^2 between the SNPs selected by each subtype-specific JIA model. Each column and row in the matrices represent one SNP in the model and SNPs are ordered by chromosome and position.

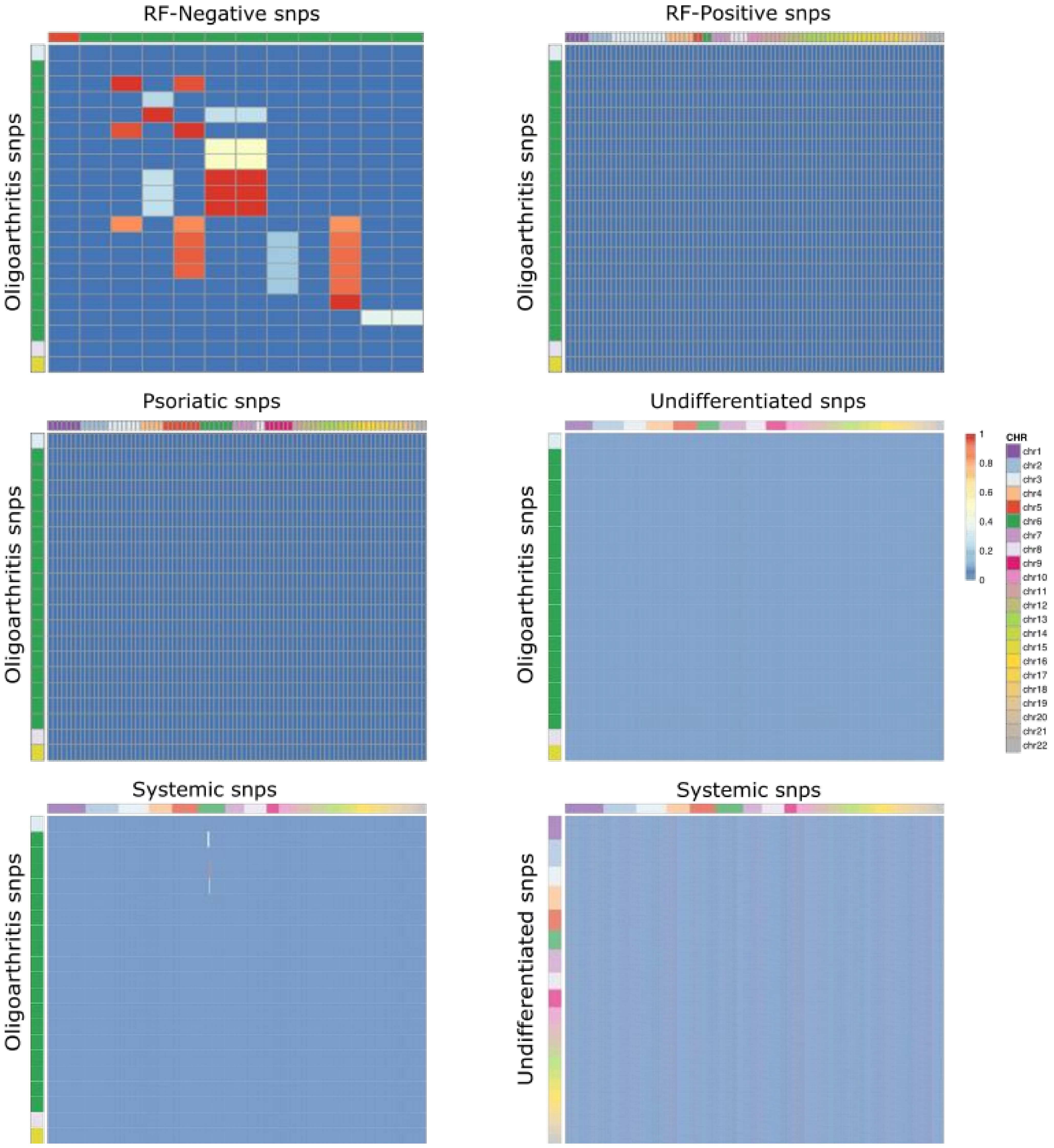

Cánovas R, *et al. Ann Rheum Dis* 2020; 79:1572–1579. doi: 10.1136/annrheumdis-2020-217421

Figure S14: r^2 between the SNPs selected by each subtype-specific JIA model. Each column and row in the matrices represent one SNP in the model and SNPs are ordered by chromosome and position.

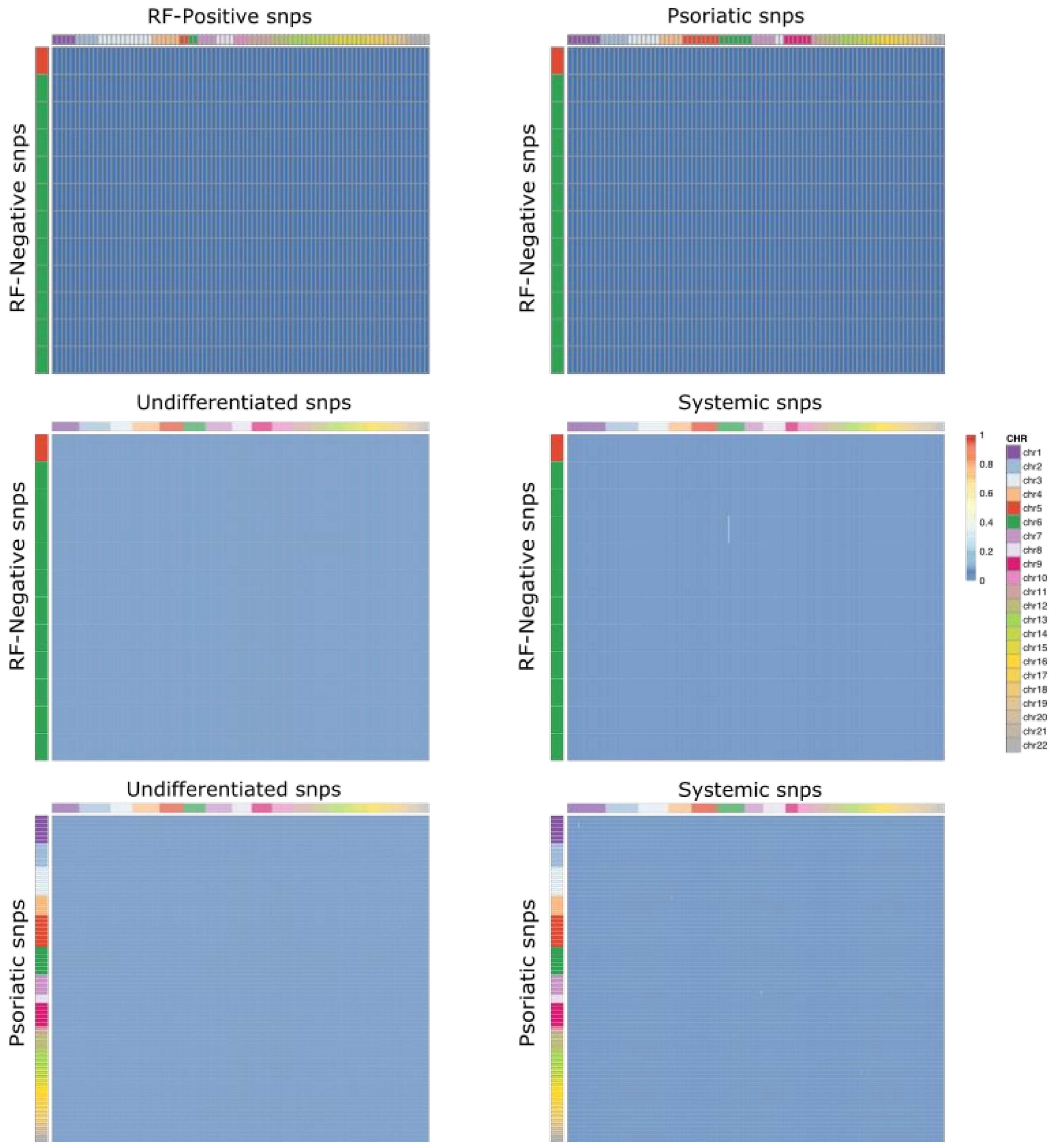

Cánovas R, *et al. Ann Rheum Dis* 2020; 79:1572–1579. doi: 10.1136/annrheumdis-2020-217421

Figure S15: r^2 between the SNPs selected by each subtype-specific JIA model. Each column and row in the matrices represent one SNP in the model and SNPs are ordered by chromosome and position.

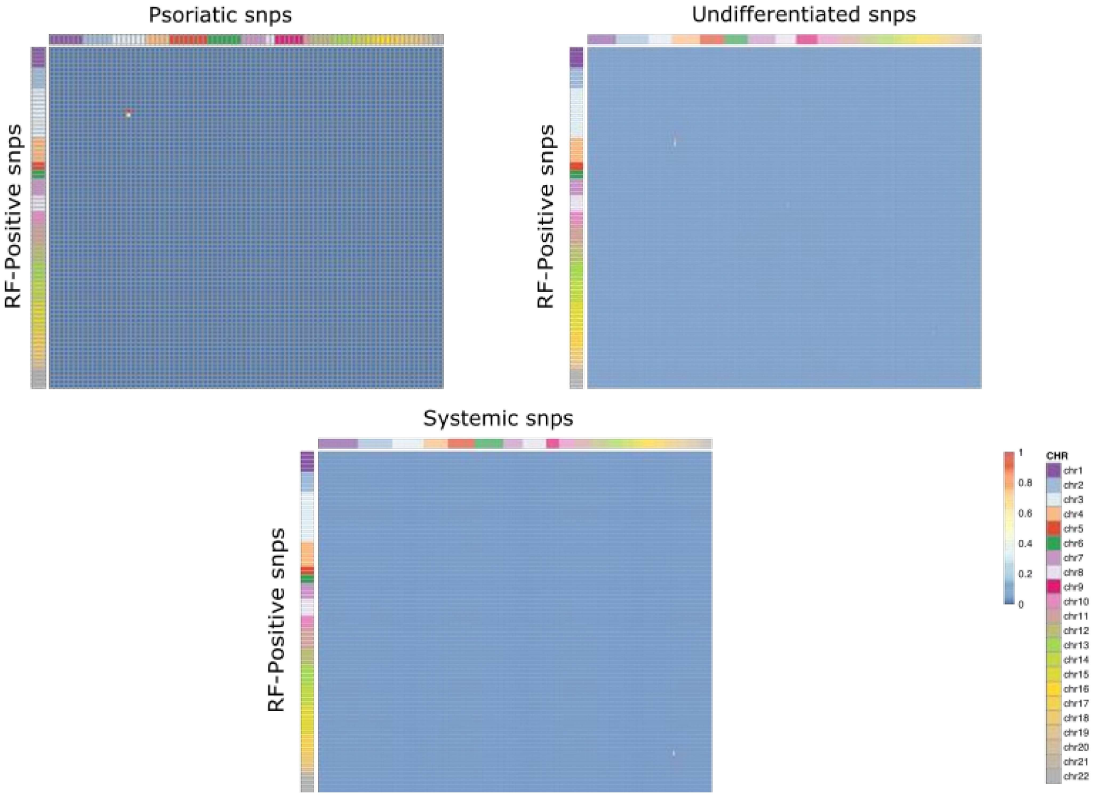

Cánovas R, *et al. Ann Rheum Dis* 2020; 79:1572–1579. doi: 10.1136/annrheumdis-2020-217421

Supplementary References

1 Hinks A, Cobb J, Marion MC, *et al.* Dense genotyping of immune-related disease regions identifies 14 new susceptibility loci for juvenile idiopathic arthritis. *Nat Genet* 2013;**45**:664–9. doi:10.1038/ng.2614

2 Abraham G, Kowalczyk A, Zobel J, *et al.* SparSNP: Fast and memory-efficient analysis of all SNPs for phenotype prediction. *BMC Bioinformatics* 2012;**13**:88. doi:10.1186/1471-2105-13-88

3 Abraham G, Malik R, Yonova-Doing E, *et al.* Genomic risk score offers predictive performance comparable to clinical risk factors for ischaemic stroke. *Nat Commun* 2019;**10**:1–10. doi:10.1038/s41467-019-13848-1

4 Inouye M, Abraham G, Nelson CP, *et al.* Genomic Risk Prediction of Coronary Artery Disease in 480,000 Adults: Implications for Primary Prevention. *J Am Coll Cardiol* 2018;**72**:1883–93. doi:10.1016/j.jacc.2018.07.079

5 Friedman J, Hastie T, Tibshirani R. Regularization paths for generalized linear models via coordinate descent. *J Stat Softw* 2010;**33**:1–22. doi:10.18637/jss.v033.i01

6 Cortes A, Hadler J, Pointon JP, *et al.* Identification of multiple risk variants for ankylosing spondylitis through high-density genotyping of immune-related loci. *Nat Genet* 2013;**45**:730–8. doi:10.1038/ng.2667

7 Evans DM, Spencer CCA, Pointon JJ, *et al.* Interaction between ERAP1 and HLA-B27 in ankylosing spondylitis implicates peptide handling in the mechanism for HLA-B27 in disease susceptibility. *Nat Genet* 2011;**43**:761–7. doi:10.1038/ng.873

8 Berntson L, Damgård M, Andersson-Gäre B, *et al.* HLA-B27 predicts a more extended disease with increasing age at onset in boys with Juvenile idiopathic arthritis. *J Rheumatol* 2008;**35**:2055–61. doi:10.1186/1546-0096-6-s1-p57

9 Robinson PC, Claushuis TAM, Cortes A, *et al.* Genetic dissection of acute anterior uveitis reveals similarities and differences in associations observed with ankylosing spondylitis. *Arthritis Rheumatol* 2015;**67**:140–51. doi:10.1002/art.38873

10 Zuber Z, Turowska-Heydel D, Sobczyk M, *et al.* Prevalence of HLA-B27 antigen in patients with juvenile idiopathic arthritis. *Reumatologia* 2015;**53**:125–30. doi:10.5114/reum.2015.53133

11 Ombrello MJ, Remmers EF, Tachmazidou I, *et al.* HLA-DRB1∗11 and variants of the MHC class II locus are strong risk factors for systemic juvenile idiopathic arthritis. *Proc Natl Acad Sci U S A* 2015;**112**:15970–5. doi:10.1073/pnas.1520779112

12 Zheng X. Imputation-based HLA typing with SNPs in GWAS studies. In: *Methods in Molecular Biology*. Humana Press Inc. 2018. 163–76. doi:10.1007/978-1-4939-8546-3_11

13 Yang J, Lee SH, Goddard ME, *et al.* GCTA: A tool for genome-wide complex trait analysis. *Am J Hum Genet* 2011;**88**:76–82. doi:10.1016/j.ajhg.2010.11.011

14 Speed D, Holmes J, Balding DJ. Evaluating and improving heritability models using summary statistics. *Nat Genet* 2020;**52**:458–62. doi:10.1038/s41588-020-0600-y

15 Bentham J, Morris DL, Cunninghame Graham DS, *et al.* Genetic association analyses implicate aberrant regulation of innate and adaptive immunity genes in the pathogenesis of systemic lupus erythematosus. *Nat Genet* 2015;**47**:1457–64. doi:10.1038/ng.3434

16 Sawcer S, Hellenthal G, Pirinen M, *et al.* Genetic risk and a primary role for cell-mediated immune mechanisms in multiple sclerosis. Nature. 2011;**476**:214–9. doi:10.1038/nature10251

17 Beecham AH, Patsopoulos NA, Xifara DK, *et al.* Analysis of immune-related loci identifies 48 new susceptibility variants for multiple sclerosis. *Nat Genet* 2013;**45**:1353–62. doi:10.1038/ng.2770

18 Tsoi LC, Spain SL, Knight J, *et al.* Identification of 15 new psoriasis susceptibility loci highlights the role of innate immunity. *Nat Genet* 2012;**44**:1341–8. doi:10.1038/ng.2467

19 Faraco J, Lin L, Kornum BR, *et al.* ImmunoChip Study Implicates Antigen Presentation to T Cells in Narcolepsy. *PLoS Genet* 2013;**9**:e1003270. doi:10.1371/journal.pgen.1003270

20 Trynka G, Hunt KA, Bockett NA, *et al.* Dense genotyping identifies and localizes multiple common and rare variant association signals in celiac disease. *Nat Genet* 2011;**43**:1193–201. doi:10.1038/ng.998

21 Onengut-Gumuscu S, Chen WM, Burren O, *et al.* Fine mapping of type 1 diabetes susceptibility loci and evidence for colocalization of causal variants with lymphoid gene enhancers. *Nat Genet* 2015;**47**:381–6. doi:10.1038/ng.3245

22 Okada Y, Wu D, Trynka G, *et al.* Genetics of rheumatoid arthritis contributes to biology and drug discovery. *Nature* 2014;**506**:376–81. doi:10.1038/nature12873

23 Stahl EA, Raychaudhuri S, Remmers EF, *et al.* Genome-wide association study meta-analysis identifies seven new rheumatoid arthritis risk loci. *Nat Genet* 2010;**42**:508–14. doi:10.1038/ng.582

24 Eyre S, Bowes J, Diogo D, *et al.* High-density genetic mapping identifies new susceptibility loci for rheumatoid arthritis. *Nat Genet* 2012;**44**:1336–40. doi:10.1038/ng.2462

25 Liu JZ, Almarri MA, Gaffney DJ, *et al.* Dense fine-mapping study identifies new susceptibility loci for primary biliary cirrhosis. *Nat Genet* 2012;**44**:1137–41. doi:10.1038/ng.2395

26 Cordell HJ, Han Y, Mells GF, *et al.* International genome-wide meta-analysis identifies new primary biliary cirrhosis risk loci and targetable pathogenic pathways. *Nat Commun* 2015;**6**. doi:10.1038/ncomms9019

27 Anderson CA, Boucher G, Lees CW, *et al.* Meta-analysis identifies 29 additional ulcerative colitis risk loci, increasing the number of confirmed associations to 47. Nat. Genet. 2011;**43**:246–52. doi:10.1038/ng.764

28 DeLong ER, DeLong DM, Clarke-Pearson DL. Comparing the Areas under Two or More Correlated Receiver Operating Characteristic Curves: A Nonparametric Approach. *Biometrics* 1988;**44**:837. doi:10.2307/2531595

Cánovas R, *et al. Ann Rheum Dis* 2020; 79:1572–1579. doi: 10.1136/annrheumdis-2020-217421
